# Cryo-EM structures of Nipah virus polymerases and high-throughput biochemical RdRp assay development enable anti-NiV drug discovery

**DOI:** 10.1063/4.0001063

**Published:** 2025-10-27

**Authors:** Ahmed Rohaim, Colin Deniston, Zhenhang Chen, Cosmo Buffolo, Tiffany Tsang, Lilli Xie, Michael DiDonato, Glen Spraggon, Matt Clifton, Nadine Jarrousse, Judith Straimer, Bo Liang

**Affiliations:** 1 Novartis, Emeryville, California, USA; 2 Novartis, San Diego, California, USA; 3 Emory University, Atlanta, Georgia, USA

## Abstract

The transcription and replication of Nipah virus (NiV) is driven by the large protein (L) and its essential co-factor phosphoprotein (P). L encodes all the viral enzymatic functions including RNA-dependent RNA polymerase (RdRp) activity while P functions as a multi-modular tetramer. To understand the mechanism of NiV polymerase and to create tools for NiV drug discovery, we studied both the full-length and truncated NiV polymerases from the Malaysia and Bangladesh strains using cryo-EM structures. We identified two key loops in the polyribonucleotidyltransferase (PRNTase) domain of L and the RdRp-PRNTase. A highly sensitive radioactive-labeled RNA synthesis assay was utilized to assess the polymerase activity of NiV. Using mutagenesis and computational tools we highlighted key residues that are paramount to viral activity. Together, these structure analyses and high-throughput biochemical assays will aid in identifying new antiviral drugs for treating the highly pathogenic henipaviruses.

